# Terminal Shoot Maturity Orchestrates LcSPL–LcFT1 Signaling to Dictate Low-Temperature Floral Induction in Litchi (*Litchi chinensis* Sonn.)

**DOI:** 10.3390/plants15182829

**Published:** 2026-09-16

**Authors:** Qiusheng Xiao, Zuanxian Su, Jiyuan Shen, Houbin Chen

**Affiliations:** 1 School of Life Sciences and Environmental Resources, Yichun University, Yichun 336000, China; 2 College of Horticulture, South China Agricultural University, Guangzhou 510642, China

**Keywords:** *Litchi chinensis*, shoot maturity, floral induction, sucrose metabolism, LcSPLs, LcFT1

## Abstract

In perennial fruit trees, erratic floral initiation and alternate bearing represent major horticultural challenges, yet how vegetative shoot maturity conditions low-temperature responsiveness remains poorly understood. Here, using litchi (*Litchi chinensis* Sonn.) as a model, we deciphered the physiological and molecular framework governing maturity-dependent floral induction under winter chilling. Mature terminal shoots exhibited exceptional flowering competence and superior inflorescence morphology, whereas immature expanding shoots failed to initiate pure panicles. Dynamic metabolic profiling revealed that shoot maturation established a high-energy status characterized by elevated sucrose accumulation and sustained transcription of trehalose-6-phosphate synthase genes (*LcTPS1/3/4*). At the transcriptional level, specific members of *LcSPL1*, *LcSPL3*, and *LcSPL10* were strongly upregulated in mature shoots, reaching peak expression at the “whitish millet” stage, marking morphological flower bud differentiation. Functional characterization in Arabidopsis thaliana confirmed that ectopic expression of *LcSPL1/3/10* significantly accelerated flowering time and reduced rosette leaf number. Mechanistically, dual-luciferase reporter assays demonstrated that LcSPL1, LcSPL3, and LcSPL10 transcriptionally activated the promoter of the florigen gene *LcFT1*, with LcSPL1 exerting the strongest transactivation, whereas the immature shoot-enriched LcSPL9 repressed *LcFT1* expression. Concurrently, mature leaves exhibited a dramatic surge in *LcFT1* transcript levels alongside persistent suppression of the floral repressor *LcTFL1*. Together, our findings demonstrate that shoot maturity confers floral competence through an integrated T6P metabolism and LcSPL1/3/10–*LcFT1* transcriptional cascade. This study provides crucial theoretical insights and potential targets for flowering-related regulation in perennial woody crops.

## 1. Introduction

Litchi (*Litchi chinensis* Sonn.), a prominent evergreen fruit tree species of the family Sapindaceae, has a long history of cultivation in tropical and subtropical regions [1,2]. However, the sustainable development of the litchi industry is severely hampered by alternate bearing, a phenomenon primarily driven by poor or erratic floral induction [3,4,5]. As a species characterized by terminal flowering, litchi undergoes floral bud differentiation in response to a complex interplay of environmental and physiological factors [2]. Although environmental cues, particularly chilling exposure during winter, are prerequisites for floral induction, the physiological status of the bearing mother shoots serves as the controllable core determining flowering success [6,7,8]. Field observations consistently demonstrate that trees with immature young flushes or ungreened red leaves prior to winter chilling fail to initiate floral buds, ultimately leading to yield loss.

Shoot maturity provides both the structural and metabolic foundation for successful floral induction. Shoot maturity was assessed based on leaf expansion, leaf color, and stem lignification status, which are widely used criteria in litchi phenological studies. Carbohydrate accumulation, particularly the dynamics of starch and soluble sugars in leaves and small twigs, is indispensable for the emergence of panicle primordia (traditionally recognized as the “whitish millet” stage) [8,9,10]. Immature leaves exhibit insufficient carbon allocation and an inability to perceive or transduce low-temperature signals. Nevertheless, precise physiological criteria for defining shoot maturity remain lacking, and the molecular mechanism explaining why young leaves inhibit floral induction remains a long-standing, unresolved question in litchi biology.

At the molecular level, plant floral transition is regulated by an integrated genetic network. The SQUAMOSA PROMOTER BINDING PROTEIN-LIKE (SPL) transcription factor family, regulated by miR156, plays a pivotal role in the age-dependent pathway governing vegetative-to-reproductive phase change [11,12]. Concurrently, upon sensing chilling signals, florigen (*FT*, *FLOWERING LOCUS T*) synthesized in mature leaves translocates long-distance through the phloem to the shoot apical meristem, where it interacts with FD (FLOWERING LOCUS D) to activate downstream floral meristem identity genes such as AP1 and LFY [13,14,15]. Although several key flowering-related genes, including *LcFT*, *LcFLC*, *LcLFY*, and *LcSPLs*, have been identified in litchi [8,16], how *LcSPL* genes modulate the floral responsiveness of terminal shoots at distinct maturity stages remains poorly understood.

To address these knowledge gaps, this study systematically analyzed the expression patterns of key floral induction genes, with a primary focus on the *LcSPL* family, in leaves of the latest shoot flush across different maturity stages. The findings aim to elucidate the physiological and molecular mechanisms by which shoot maturity governs floral transition, thereby providing theoretical insights and molecular targets for the regulation of flowering in woody perennial fruit crops.

## 2. Results

### 2.1. Terminal Shoot Maturity Influences Litchi Floral Induction

**Figure 1:**
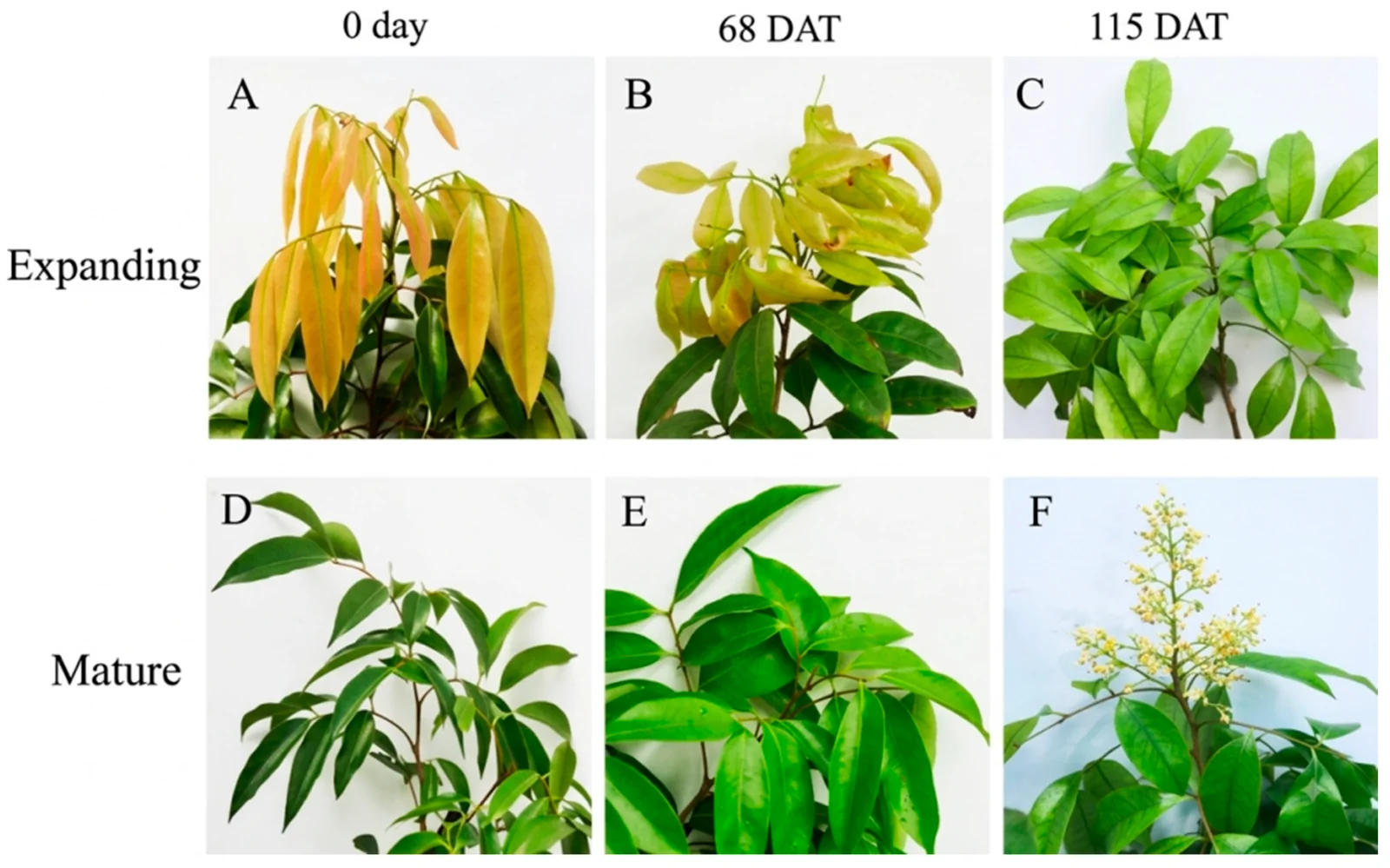
Floral induction status of litchi terminal shoots at different maturity stages following low-temperature treatment: (**A**–**C**) Morphology of terminal shoots at the leaf-expanding stage prior to low-temperature treatment (day 0), at the “whitish millet” stage, and at the blooming stage, respectively. (**D**–**F**) Morphology of mature terminal shoots at the corresponding time points. DAT, days after treatment.

Litchi inflorescences originate from terminal shoot apical meristems (SAMs), which also govern recurrent vegetative flushes. To evaluate how terminal shoot maturity conditions affect floral development, potted litchi trees possessing terminal flushes at either the leaf-expanding or mature stage (Figure 1A,D) were exposed to a chilling regime (15/10 °C, 12 h light/12 h dark). Following 60 days of cold induction, the trees were returned to ambient temperature (25 °C). Shoots that were mature prior to treatment achieved floral initiation within 8 days (Figure 1E) and ultimately developed full panicles (Figure 1F). Conversely, shoots treated during the expansion stage failed to initiate flowering (Figure 1B,C).

To evaluate the influence of shoot maturity on litchi floral induction, the flowering characteristics of terminal shoots at the expanding and mature stages were quantified following low-temperature treatment (Table 1). The time required to reach the “whitish millet” stage showed no statistically significant difference between the expanding and mature shoots. However, shoot maturity markedly enhanced both flowering efficiency and inflorescence morphology. The flowering rate of mature terminal shoots reached 84.62%, which was significantly higher than that of expanding shoots, at 14.51%. Notably, while expanding shoots failed to form pure flowering shoots, mature shoots exhibited a pure flowering rate of 62.22% (*p* < 0.01). Furthermore, the inflorescence dimensions were significantly superior in mature shoots compared to expanding ones, displaying nearly double the length and more than twice the width.

### 2.2. Dynamic Changes in Sugar Accumulation During Low-Temperature Floral Induction

**Figure 2:**
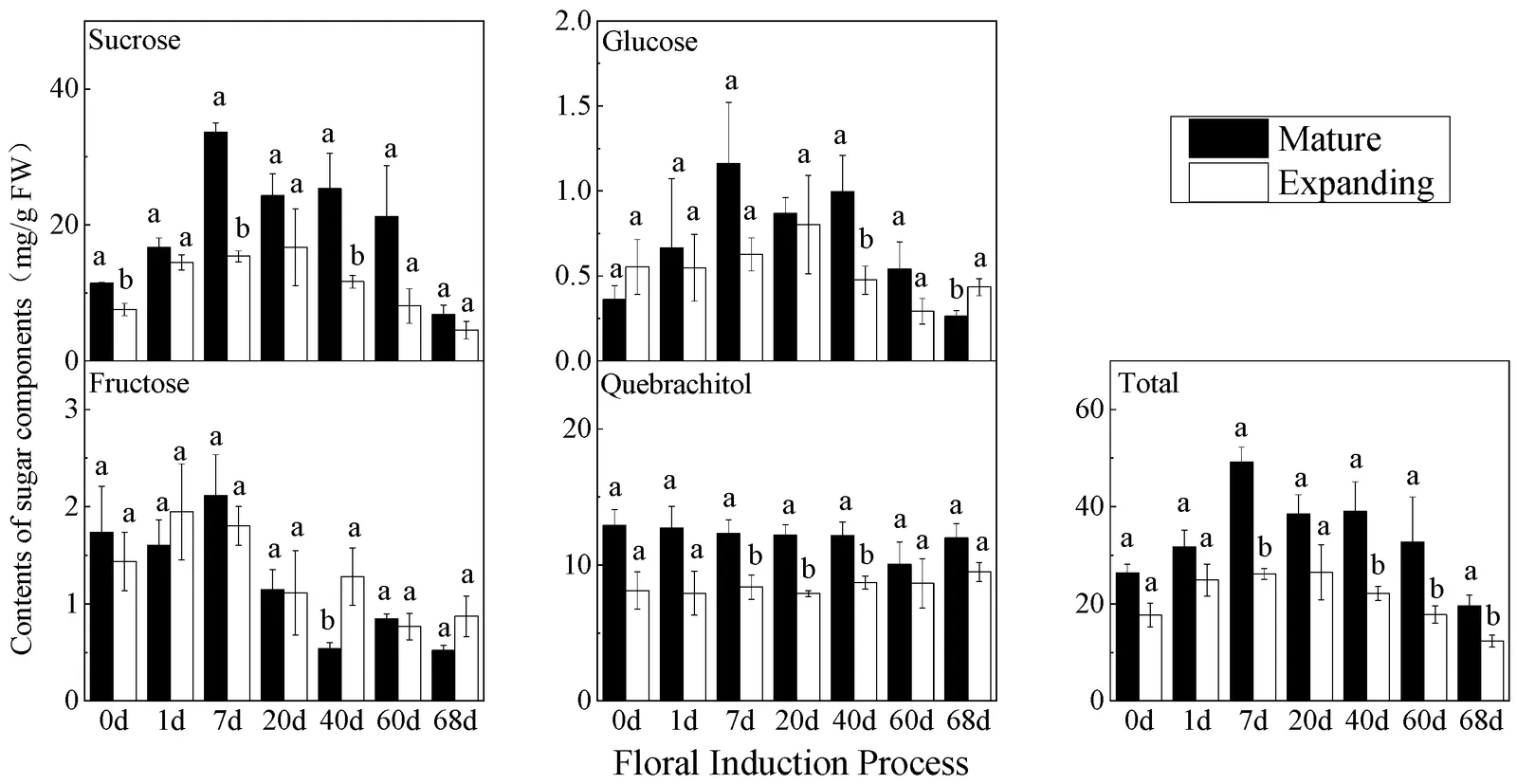
Dynamic changes in soluble sugar content in terminal shoots of different maturity stages during low-temperature floral induction. Data are presented as mean ± SE (*n* = 3). Different lowercase letters above the bars indicate significant differences between mature and expanding shoots at the same time point (*p* < 0.05, Student’s *t*-test). FW, fresh weight.

To clarify the metabolic differences between shoot maturity levels during floral induction, dynamic changes in primary soluble sugars (sucrose, glucose, fructose, quebrachitol, and total sugar) were monitored across a 68-day chilling period (Figure 2). Overall, mature terminal shoots maintained significantly higher levels of sugar accumulation compared to expanding shoots throughout the low-temperature treatment. Sucrose was the primary soluble sugar; in mature shoots, sucrose and total sugars peaked rapidly at 7 d, remaining significantly higher than in expanding shoots from 7 d to 60 d (*p* < 0.05). By 68 d, both groups declined to similarly low levels. Glucose in mature shoots peaked at 7 d and significantly exceeded expanding shoots at 20 d and 40 d. Fructose remained generally low, though mature shoots showed significantly lower levels than expanding shoots at 40 d.

### 2.3. Expression Analysis of LcTPS Genes in Leaves of the Terminal Shoot at Different Maturity Stages

**Figure 3:**
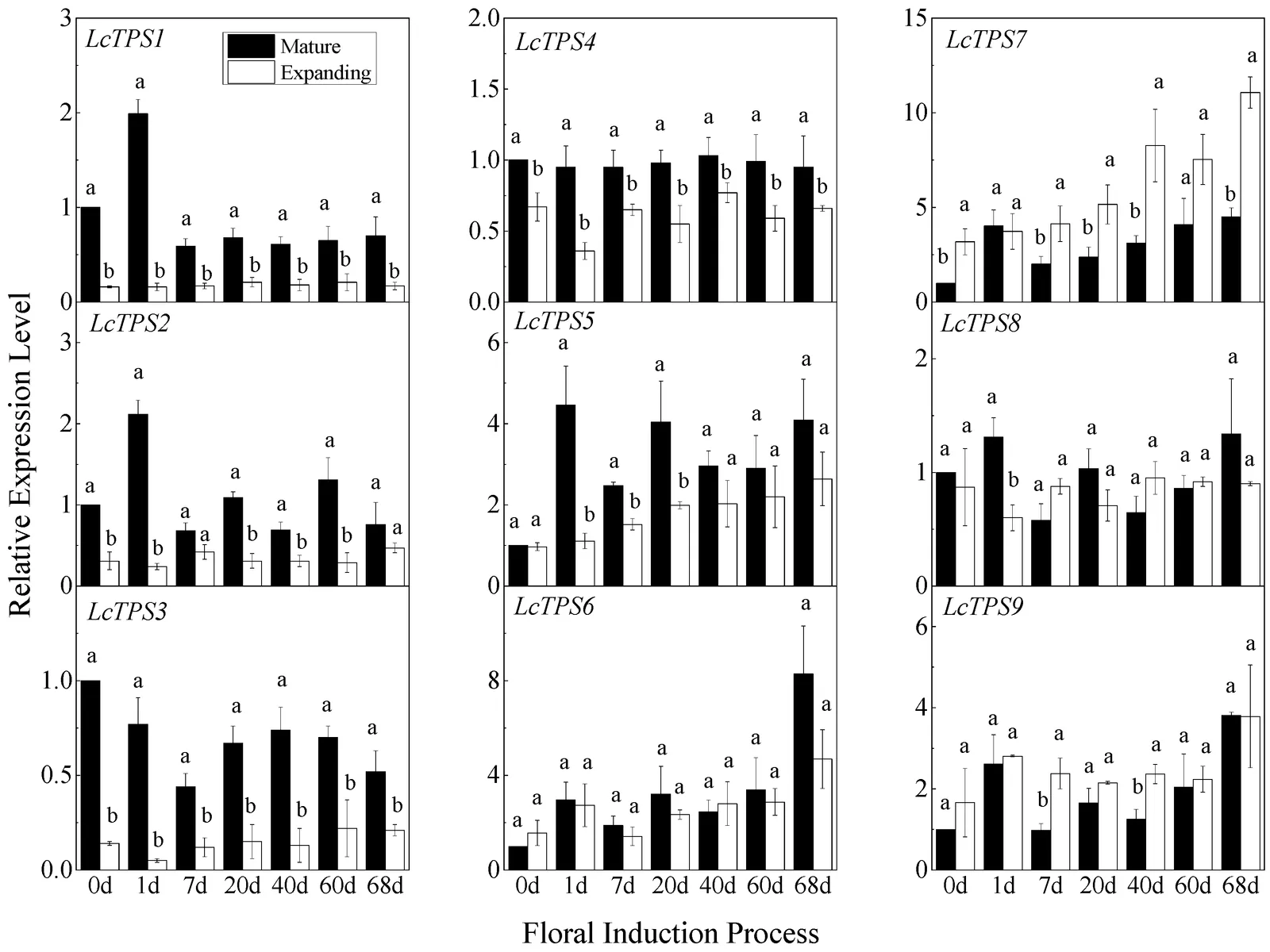
Expression patterns of LcTPS family genes in litchi terminal shoots of different maturity stages during low-temperature floral induction. Different lowercase letters indicate significant differences at *p* < 0.05.

To evaluate the involvement of trehalose-6-phosphate signaling in litchi floral transition, temporal expression profiles of nine LcTPS family members were tracked in mature and expanding terminal shoots during chilling treatment (Figure 3). Distinct transcription kinetics were observed between the two maturity stages. In mature shoots, LcTPS1, LcTPS2, and LcTPS5 functioned as early responders, undergoing rapid upregulation within 1 d of chilling and maintaining significantly higher expression levels than in expanding shoots throughout cold exposure (*p* < 0.05). Likewise, LcTPS3 and LcTPS4 displayed persistently elevated transcript abundance in mature shoots across the entire 68-day induction period, whereas LcTPS3 remained near baseline levels in expanding shoots. Conversely, LcTPS7 was enriched in expanding shoots, showing significantly higher expression at 20, 40, and 68 d, while LcTPS8 maintained comparable transcript levels across maturity stages. Crucially, at the onset of the “whitish millet” stage, a vital physiological marker of morphological flower bud differentiation, mature shoots exhibited significantly elevated transcript levels of LcTPS1, LcTPS3, and LcTPS4 relative to expanding shoots. This preferential accumulation of LcTPS1, LcTPS3, and LcTPS4 transcripts in mature shoots closely aligns with their high flowering competence, indicating that the sustained activation of these core genes serves as a pivotal molecular driver promoting litchi floral induction.

### 2.4. Expression Analysis of LcSPL Genes in Leaves of the Terminal Shoot at Different Maturity Stages

**Figure 4:**
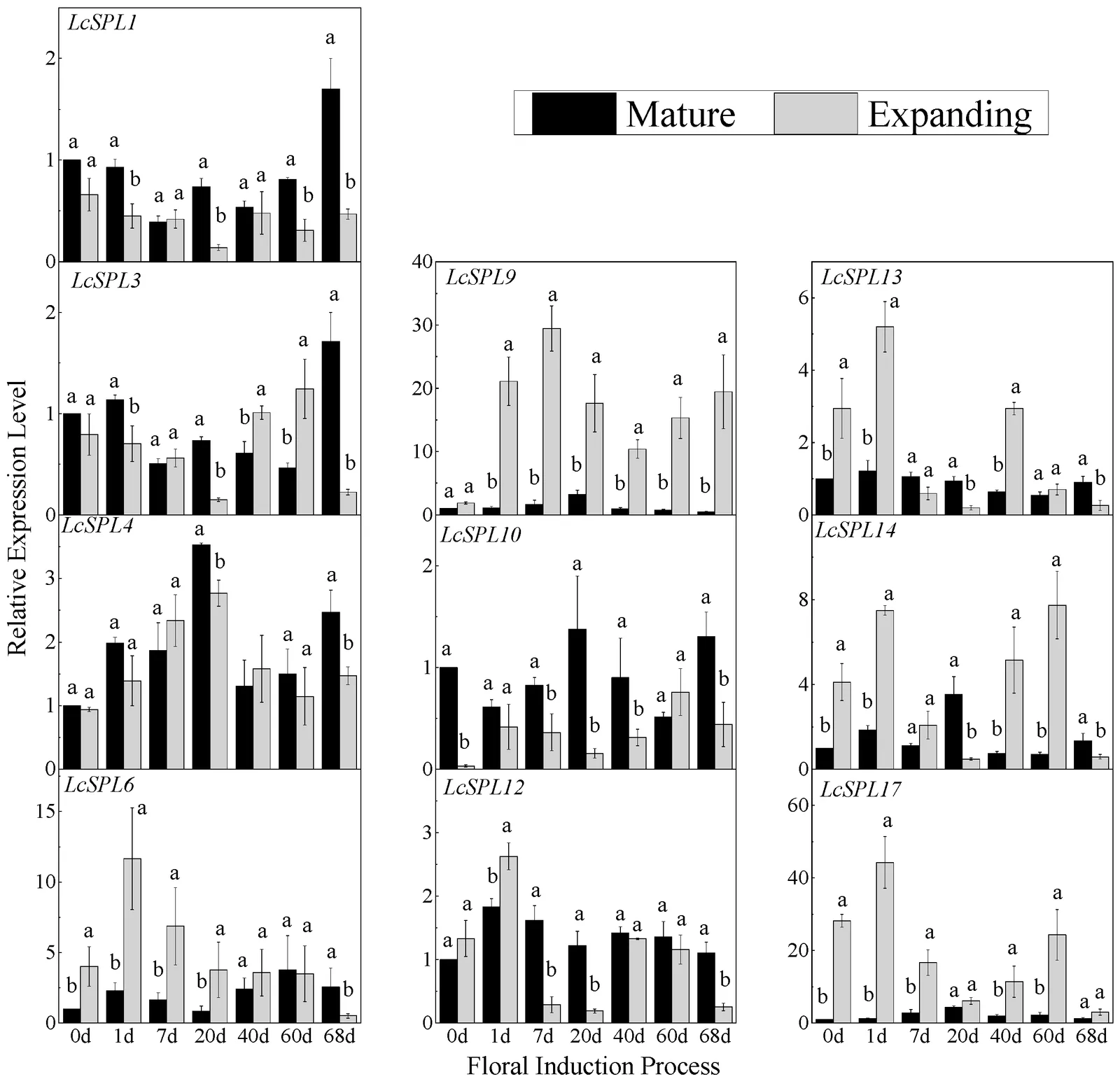
Expression patterns of *LcSPL* family genes in litchi terminal shoots of different maturity stages during low-temperature floral induction. Different lowercase letters indicate significant differences at *p* < 0.05.

To evaluate the involvement of the microRNA156/SQUAMOSA PROMOTER BINDING PROTEIN-LIKE module in litchi flowering, temporal expression profiles of *LcSPL* family members were tracked in mature and expanding terminal shoots across the chilling period up to the “whitish millet” stage (Figure 4). Crucially, key flower-promoting genes *LcSPL1*, *LcSPL3*, *LcSPL4*, and *LcSPL10* exhibited significantly higher transcript abundance in mature leaves compared to expanding ones at the critical “whitish millet” stage (*p* < 0.05). At this physiological landmark marking the onset of flower bud differentiation, these four genes were strongly upregulated and reached peak or near-peak levels in mature shoots; specifically, *LcSPL4* and *LcSPL10* maintained elevated baseline expression throughout mid-to-late chilling, whereas *LcSPL1* and *LcSPL3* displayed a distinct late-stage accumulation precisely coinciding with “whitish millet” formation. This preferential enrichment of *LcSPL1/3/4/10* in mature leaves strongly correlates with their high flowering competence, indicating that upregulation of these specific *LcSPL* members at the “whitish millet” stage serves as a key molecular driver promoting litchi floral transition. In contrast, *LcSPL6*, *LcSPL9*, *LcSPL13*, *LcSPL14*, and *LcSPL17* maintained substantially higher expression in expanding shoots across nearly all sampled time points, with prominent transcript peaks during early chilling, while *LcSPL12* displayed a stage-specific shift from higher early expression in expanding shoots to significantly elevated levels in mature shoots during later stages, including at the “whitish millet” phase.

### 2.5. Expression Analysis of LcSPL Genes in Leaves of Seedling Plants at Different Maturity Stages

**Figure 5:**
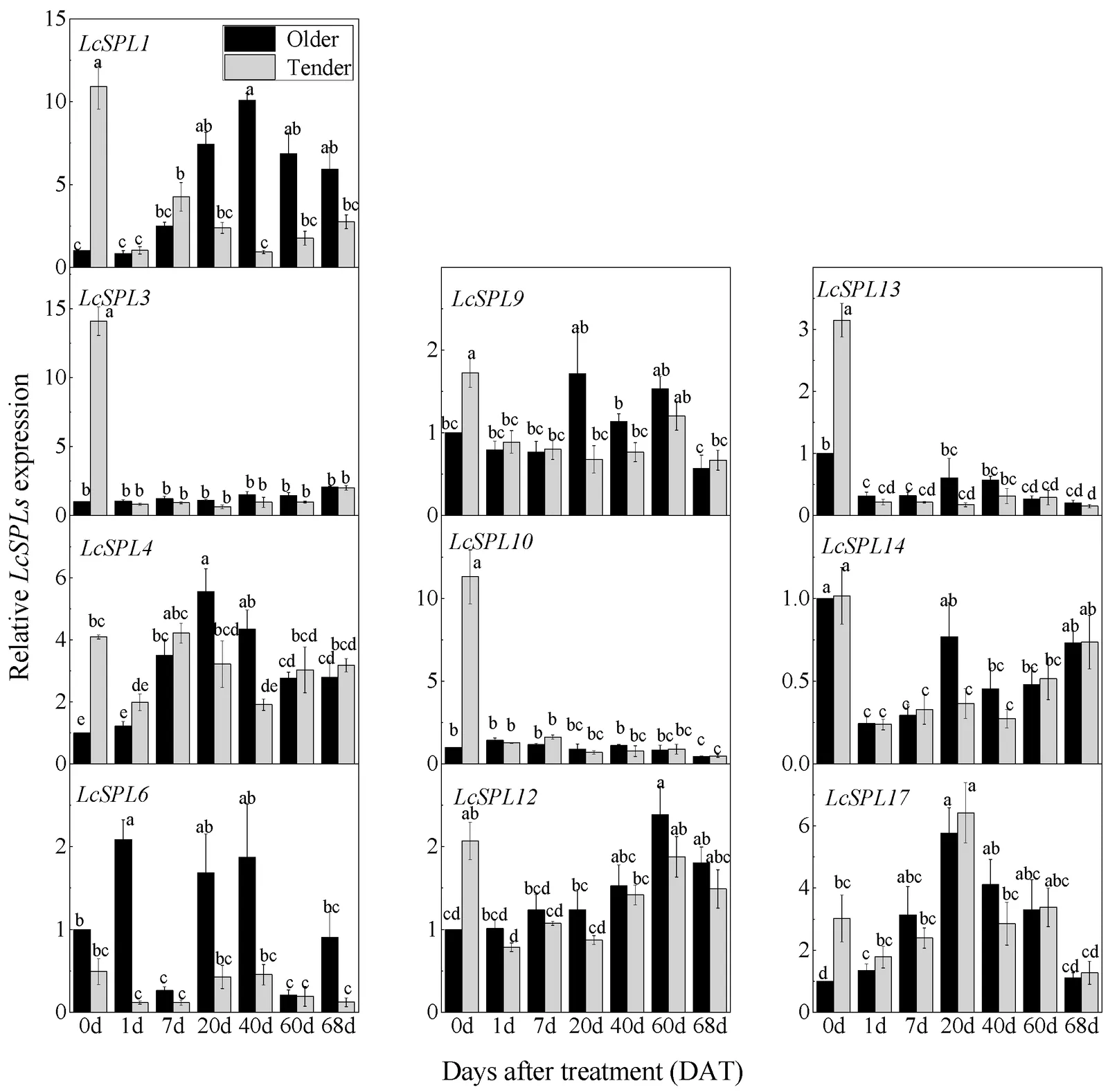
Expression analysis of *LcSPLs* in leaves of the seedlings under assumed floral induction conditions (15/10 °C, 60 d). Tender means the young leaf on the top of the seedlings, and Older means the secondary old leaf; different lowercase letters indicate significant differences at the *p* < 0.05 level.

Under simulated low-temperature floral induction conditions, members of the *LcSPL* gene family displayed distinct leaf-maturity-dependent expression dynamics in litchi seedlings (Figure 5). Crucially, *LcSPL1*, *LcSPL4*, and *LcSPL6* maintained significantly higher transcript abundance in secondary older leaves than in tender young leaves during mid-to-late chilling, with *LcSPL1* and *LcSPL4* exhibiting peak expression at 20 d in older leaves. Conversely, *LcSPL3*, *LcSPL10*, and *LcSPL13* showed sharp, transient expression peaks in tender young leaves at 0 d prior to cold exposure, followed by rapid downregulation upon entering simulated chilling conditions. Meanwhile, *LcSPL12* and *LcSPL17* were progressively induced in both leaf types during late chilling, reaching maximum transcript levels at 60 d and 20 d, respectively (*p* < 0.05).

### 2.6. Expression Analysis of Key Genes Involved in Floral Induction in Leaves of the Terminal Shoot at Different Maturity

**Figure 6:**
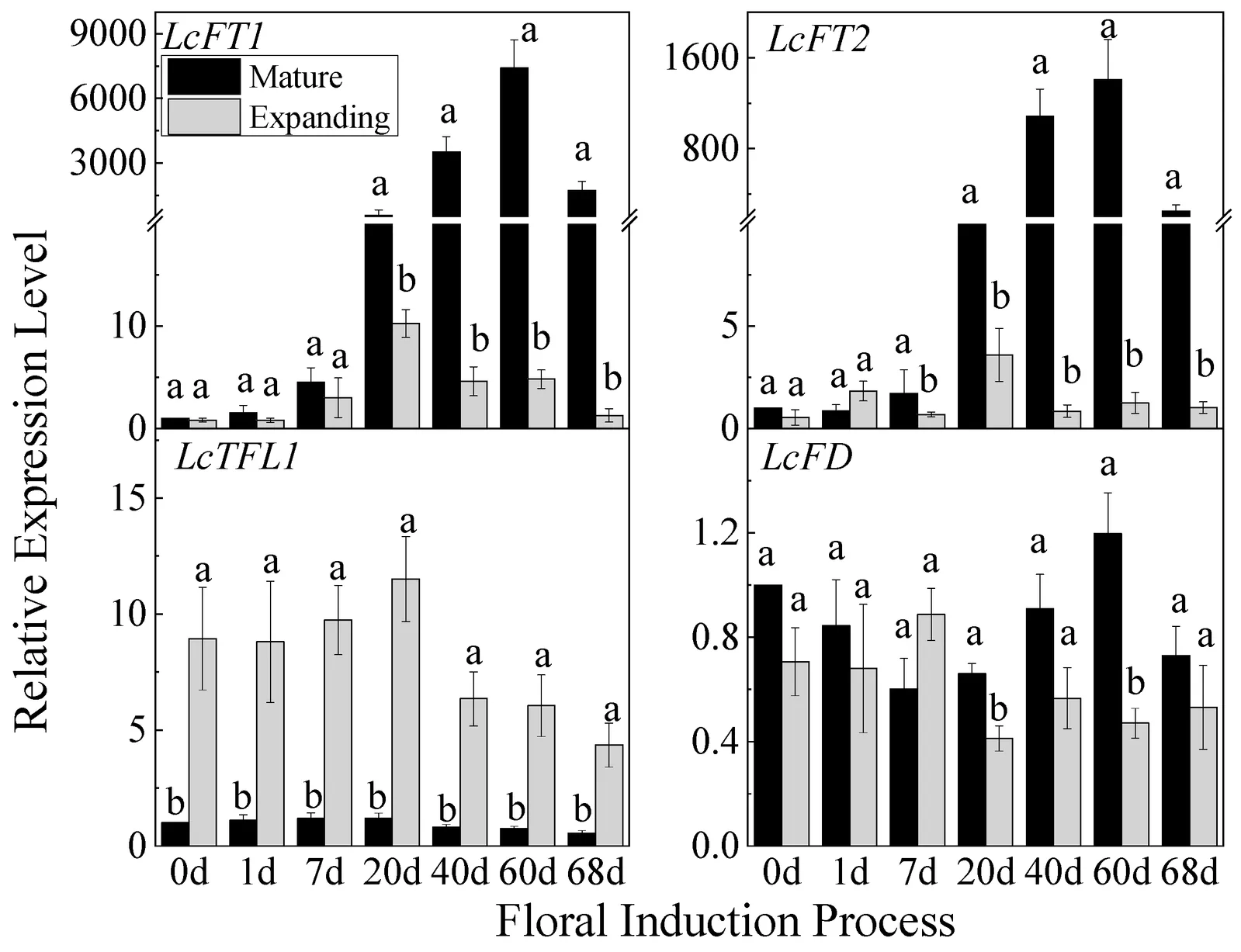
Expression patterns of florigen and anti-florigen genes in litchi terminal shoots of different maturity stages during low-temperature floral induction. Different lowercase letters indicate significant differences at *p* < 0.05.

To elucidate the florigen-mediated regulation of litchi floral induction, the temporal expression profiles of *LcFT1*, *LcFT2*, *LcTFL1*, and *LcFD* were monitored in mature and expanding terminal shoots throughout chilling up to the “whitish millet” stage (Figure 6). Crucially, *LcFT1* emerged as the primary molecular driver of litchi floral initiation. While both *LcFT1* and *LcFT2* remained at baseline levels during early chilling, *LcFT1* exhibited a massive, predominant upregulation in mature shoots starting at 20 d and reached an unprecedented peak at 60 d. Throughout mid-to-late chilling (20–68 d), *LcFT1* transcript abundance in mature leaves was extraordinarily higher than in expanding shoots (*p* < 0.05), where it peaked at only approximately 10-fold; even at the “whitish millet” stage, *LcFT1* maintained exceptionally elevated expression in mature shoots, directly aligning with their superior flowering competence. Although *LcFT2* displayed a similar dynamic trend, peaking at over 1400-fold at 60 d, its expression magnitude remained substantially lower than that of *LcFT1*, while the florigen interaction partner *LcFD* maintained relatively stable expression overall, with significantly higher levels in mature shoots at 20 d and 60 d. In stark contrast to *LcFT1*, the floral repressor *LcTFL1* remained persistently high in expanding shoots across the entire 68 d period while being almost completely suppressed in mature shoots, further emphasizing that the dramatic transcript enrichment of *LcFT1* over *LcTFL1* in mature leaves provides the decisive florigenic signal governing litchi floral induction.

### 2.7. Transcriptional Activation and Repression of the LcFT1 Promoter by LcSPL Transcription Factors

**Figure 7:**
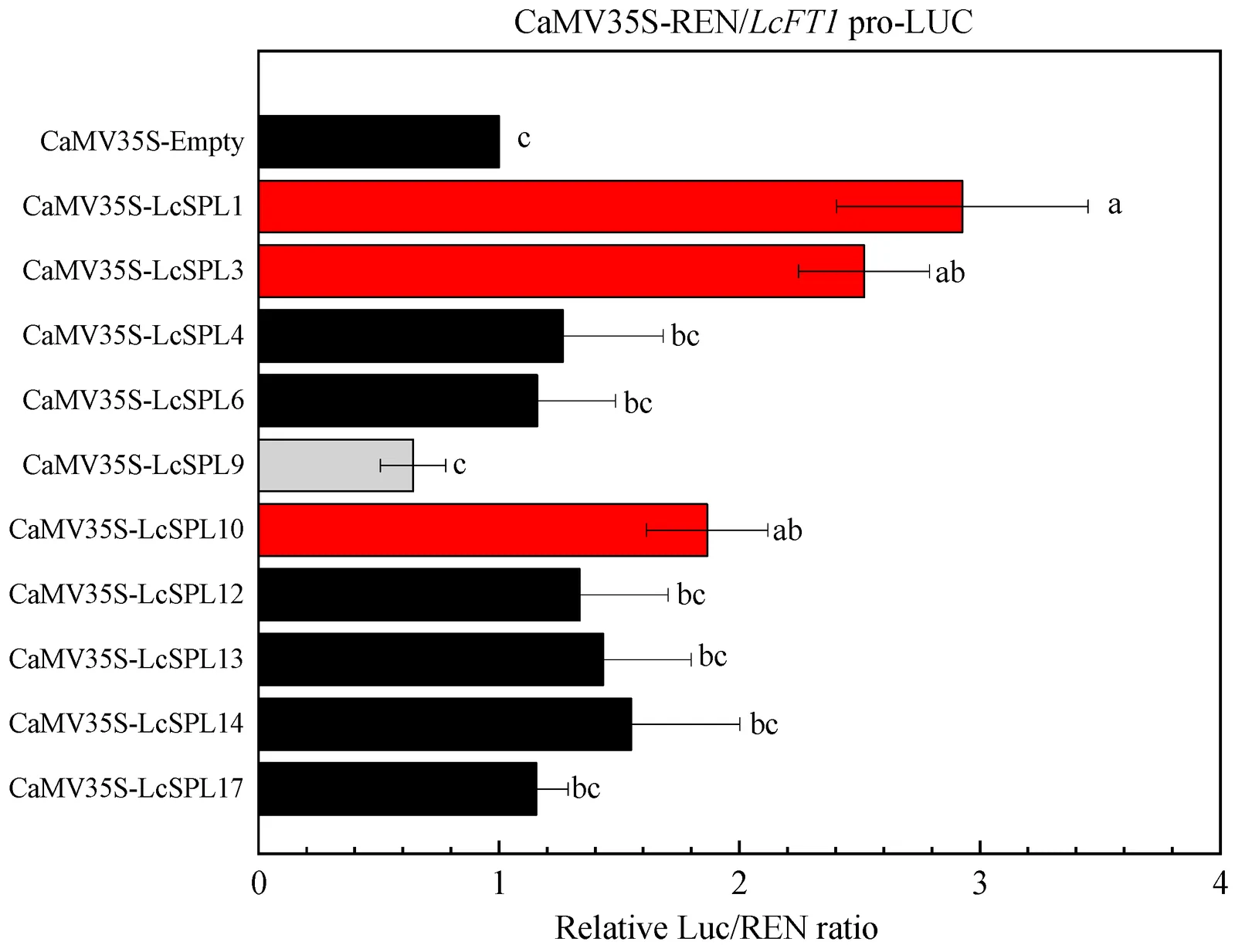
Analysis of interaction of LcSPLs and the promoter of *LcFT1* by dual-luciferase complementation assay. Transient coexpression of LcSPLs and the promoter of *LcFT1* was assessed in tobacco (*N. benthamiana*) leaves. The ratio of firefly luciferase (LUC) to renilla luciferase (REN) of the empty vector (SK) plus promoter was set at 1; red bars indicate transcriptional activation of the LcFT1 promoter, while grey bars indicate transcriptional repression. Data are presented as means ± SD (*n* = 3), and significant differences are indicated with different letters.

To evaluate the transcriptional regulation of the core florigen gene *LcFT1* by LcSPL transcription factors, a dual-luciferase reporter assay was performed in Nicotiana benthamiana leaves (Figure 7). Crucially, LcSPL1, LcSPL3, and LcSPL10 operated as key transcriptional activators of *LcFT1*, with LcSPL1 driving the highest transactivation level (*p* < 0.05), followed by LcSPL3 and LcSPL10. Conversely, LcSPL9 exhibited a significant repressive effect on the *LcFT1* promoter, suppressing the relative LUC/REN ratio down to 0.63. Other evaluated members (LcSPL4, 6, 12, 13, 14, and 17) showed no significant regulatory effects relative to the control. These results demonstrate that LcSPL1, LcSPL3, and LcSPL10 promote litchi flowering through transcriptional activation of the *LcFT1* promoter, whereas LcSPL9 functions as a potential transcriptional repressor.

### 2.8. Effects of LcSPL1/3/4/9/10 on Flowering in Arabidopsis

To evaluate the functional roles of *LcSPL1*, *LcSPL3*, *LcSPL9*, and *LcSPL10* in regulating flowering time, T2 transgenic Arabidopsis plants overexpressing these individual genes (35S::*LcSPL*) were analyzed alongside wild-type (WT, Col-0) controls (Table 2). Ectopic expression of *LcSPL1*, *LcSPL3*, and *LcSPL10* significantly accelerated the floral transition, with 35S::*LcSPL1*, 35S::*LcSPL3*, and 35S::*LcSPL10* lines exhibiting markedly earlier flowering than WT plants. Consistently, these transgenic lines produced significantly fewer rosette leaves at flowering compared to WT, confirming that ectopic expression of *LcSPL1*, *LcSPL3*, or *LcSPL10* confers a distinct early-flowering phenotype in Arabidopsis. In contrast, 35S::*LcSPL9* plants exhibited no significant differences in flowering time and rosette leaf number at flowering relative to WT.

**Figure 8:**
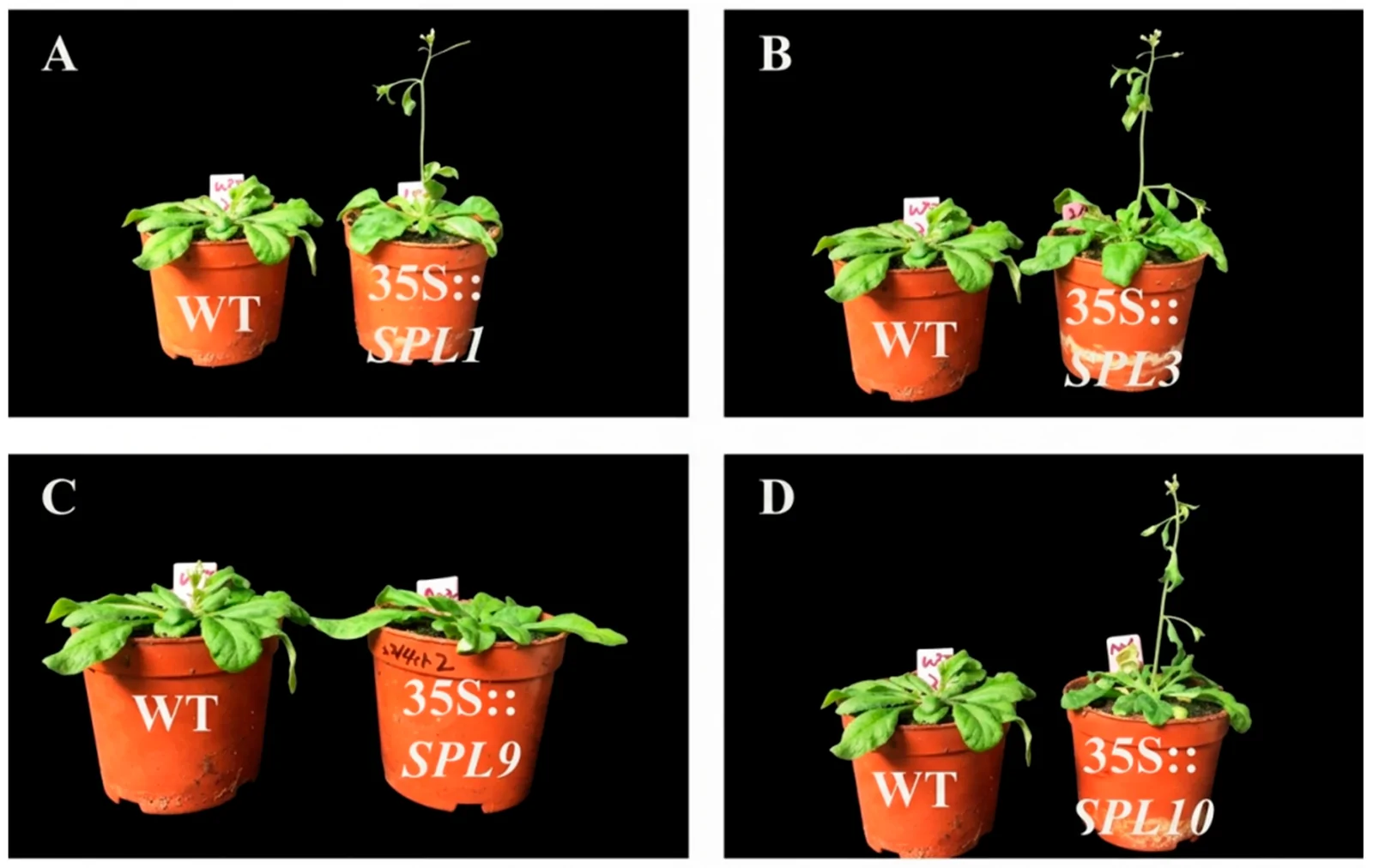
Phenotypic characterization of transgenic Arabidopsis overexpressing *LcSPL1/3/9/10*. Flowering phenotypes of homozygous T3 transgenic lines ectopically overexpressing *LcSPL1* (**A**), *LcSPL3* (**B**), *LcSPL9* (**C**), and *LcSPL10* (**D**) compared with wild-type (WT, Col-0) plants grown under long-day conditions.

Visual phenotypic observation confirmed marked differences in flowering time among the distinct overexpression lines (Figure 8). Ectopic expression of *LcSPL1* (Figure 8A), *LcSPL3* (Figure 8B), and *LcSPL10* (Figure 8D) consistently conferred a distinct early-flowering phenotype; while wild-type (WT) controls remained strictly in the vegetative rosette stage without visible inflorescence bolting, the 35S::*LcSPL1*, 35S::*LcSPL3*, and 35S::*LcSPL10* transgenic lines had already bolted, elongated main floral stems, formed open flower buds, and initiated this transition with notably fewer rosette leaves. In contrast, 35S::LcSPL9 overexpressing plants (Figure 8C) exhibited no observable difference in growth stage or flowering time relative to WT, remaining in the vegetative rosette phase without signs of premature bolting.

## 3. Discussion

Floral induction in perennial fruit trees is a pivotal developmental transition governed by a complex interplay between seasonal environmental signals and internal physiological competency [17,18,19,20]. In litchi, erratic floral initiation and alternate bearing pose severe challenges to sustainable production. Although winter chilling is a prerequisite environmental trigger, field management often encounters a major bottleneck: trees exhibiting immature terminal shoots during the chilling window fail to initiate flower buds (Figure 1), leading to vegetative flush emergence instead [2,21,22,23]. Despite its horticultural significance, the physiological and molecular framework explaining why shoot maturity conditions low-temperature responsiveness has remained elusive.

In this study, by integrating physiological assays, metabolic profiling, qRT-PCR expression dynamics, dual-luciferase reporter assays, and ectopic Arabidopsis transformation, we established that terminal shoot maturity orchestrates carbohydrate accumulation, T6P signaling, and an LcSPL1/3/10–*LcFT1* transcriptional cascade to overcome LcTFL1-mediated floral repression, thereby conferring floral competence.

Carbohydrates act as essential energy sources and primary signals during floral transition [9,24,25]. Mature terminal shoots maintained significantly higher sucrose and total sugar levels than expanding shoots throughout chilling, establishing a high-energy state (Figure 2). Expanding shoots, acting as active carbon sinks, failed to accumulate equivalent reserves [8]. *TPS* acts as a primary sucrose sensor that regulates flowering [26,27,28,29]. *LcTPS1*, *LcTPS2*, and *LcTPS5* acted as rapid cold-responders in mature leaves, maintaining elevated expression throughout chilling. Crucially, *LcTPS1*, *LcTPS3*, and *LcTPS4* were strongly enriched in mature leaves at the “whitish millet” stage (Figure 3). This maturity-dependent activation of the T6P pathway directly aligns with the high flowering rate in mature shoots compared to expanding shoots, proving that carbohydrate saturation provides the metabolic permissive signal for floral transition.

Expression analysis revealed distinct functional diversification among *LcSPL* genes based on shoot maturity [8,16,30]. *LcSPL1*, *LcSPL3*, *LcSPL4*, and *LcSPL10* were preferentially enriched in mature leaves of adult trees and older seedling leaves, reaching peak expression at the “whitish millet” stage (Figure 4). Conversely, *LcSPL6/9/13/14/17* were predominantly expressed in expanding shoots and young seedling leaves (Figure 5). Ectopic overexpression in Arabidopsis confirmed these distinct roles: 35S::LcSPL1/3/10 transgenic lines displayed pronounced early-flowering phenotypes with reduced rosette leaf numbers, whereas 35S::*LcSPL9* lines showed no acceleration of flowering (Figure 8). These results establish *LcSPL1/3/10* as direct genetic promoters of phase change requiring shoot maturity for induction.

In addition to *LcSPL1*, *LcSPL3*, and *LcSPL10*, *LcSPL4* was also significantly upregulated in mature shoots at the “whitish millet” stage, suggesting that it may also be associated with shoot maturation and floral competence. However, unlike LcSPL1/3/10, LcSPL4 did not significantly transactivate the *LcFT1* promoter in the dual-luciferase assay, implying that LcSPL4 may function through an FT-independent mechanism. It is therefore plausible that the high expression of LcSPL4 in mature shoots represents an additional regulatory layer that supports floral transition independently of the LcFT1 pathway. Alternatively, LcSPL4 may act cooperatively with LcSPL1/3/10 through protein–protein interactions to enhance downstream gene activation. Further functional characterization, including overexpression of LcSPL4 in Arabidopsis and genome-wide identification of its downstream targets, will be necessary to clarify the precise role of LcSPL4 in flowering regulation.

It is noteworthy that although LcSPL9 repressed the *LcFT1* promoter in the dual-luciferase assay, overexpression of *LcSPL9* in Arabidopsis did not result in delayed flowering (Table 2). This apparent discrepancy may be explained by several factors. First, the repression of *LcFT1* by LcSPL9 was observed in a heterologous transient expression system, which may not fully recapitulate the native regulatory environment of Arabidopsis; the endogenous *FT* promoter may not be a direct target of LcSPL9, or its repression may require litchi-specific cofactors or post-translational modifications that are absent in Arabidopsis. Second, functional divergence among *SPL* genes between litchi and Arabidopsis may lead to species-specific outcomes; LcSPL9 might function as a flowering repressor in specific litchi tissues or developmental contexts, whereas its ectopic expression in Arabidopsis may be insufficient to exert a strong effect. Collectively, these possibilities suggest that the repressive function of LcSPL9 on *LcFT1* is context-dependent and may not be directly transferable across species. Further studies, including tissue-specific expression analysis in litchi and identification of LcSPL9-interacting proteins, are needed to clarify its exact role in flowering regulation.

*FT* and *TFL1* balance determines SAM fate during chilling [31,32,33,34,35]. In mature leaves, *LcFT1* transcript levels surged dramatically under cold exposure and remained high at the “whitish millet” stage, whereas expanding shoots exhibited lower *LcFT1* induction alongside persistent elevation of the repressor *LcTFL1* (Figure 6). Dual-luciferase reporter assays provided the direct mechanism linking maturity to florigen activation: LcSPL1/3/10 significantly activated the *LcFT1* promoter activity (Figure 7), with LcSPL1 exerting the strongest transactivation, whereas LcSPL9 repressed *LcFT1* [8,36].

In summary, expanding shoots remain in a vegetative state due to low sugar reserves, low *LcTPS1/3/4* expression, and high *LcTFL1* and *LcSPL9* levels. Shoot maturation triggers sucrose accumulation and T6P signaling, upregulating *LcSPL1/3/10*. These LcSPLs transcriptionally activated *LcFT1* expression, shifting the *LcFT1/LcTFL1* ratio to promote panicle initiation in response to winter chilling. Furthermore, it is worth noting that temperature effects on plant flowering are not limited to the regulation of flowering time; temperature variation can also modify floral morphology. Researchers have found that in a special rapeseed line, the number of pistils per flower, as well as the consequent number of pods, can be changed by temperature, which is related to rapeseed yield [37,38]. In litchi, although the present study focused on the timing of floral initiation and the underlying sugar–SPL–FT regulatory module, the potential effects of low-temperature induction on floral organ development and panicle architecture remain largely unexplored. Whether shoot maturity and temperature interact to influence floral morphology and subsequent fruit set in litchi is an interesting question that warrants further investigation. Addressing this issue may provide additional strategies for improving yield stability in litchi production.

## 4. Materials and Methods

### 4.1. Plant Materials and Treatments

‘Guiwei’ is a well-known mid-season cultivar originating from Guangdong Province, China. It was selected from open-pollinated seedlings, and its exact pedigree is not fully documented, although it is widely cultivated in southern China. Phenologically, ‘Guiwei’ typically flowers in March to April, and fruits mature from late June to early July in Guangdong. The fruit is nearly round with bright red skin and prominent protuberances; the flesh is milky white, crisp, and sweet with a distinctive osmanthus-like aroma. To evaluate low-temperature-induced flowering, uniform and vigorous three-year-old air-layered ‘Guiwei’ litchi potted trees were selected. The experimental trees were grown at the South China Agricultural University, Guangzhou, Guangdong, China (23°09′ N, 113°21′ E). Mature terminal shoots were defined as those with fully expanded, dark green, leathery leaves and lignified, brownish stems that had ceased elongation; expanding terminal shoots were defined as those with light green, soft, developing leaves and non-lignified, greenish stems still undergoing active elongation. Four trees with the terminal flush at the expanding and mature stages were subjected to low-temperature treatment (15/10 °C, day/night) in a temperature-controlled growth room for 60 days, followed by transfer to ambient conditions (25/20 °C, day/night) to promote floral initiation. Each tree served as an independent biological replicate using a randomized sampling design. Leaf samples from the terminal flush were collected at seven time points: 0, 1, 7, 20, 40, and 60 days during low-temperature treatment, as well as at the “whitish millet” stage. Collected leaves were immediately flash-frozen in liquid nitrogen and stored at −80 °C for subsequent assays.

For the simulated low-temperature induction experiment on juvenile plants, 20 uniform and vigorous one-year-old litchi seedlings were transferred into an artificial climate chamber and maintained under low temperature (15/10 °C, day/night) for 60 days before being moved to ambient temperature (25/20 °C, day/night). Leaves of two distinct maturity levels—apical tender leaves and basal older leaves—were harvested at seven time points: 0, 1, 7, 20, 40, 60, and 68 days after treatment initiation. Sample collection and preservation procedures strictly followed the protocol described for adult potted trees.

### 4.2. Floral Biology Statistics of Litchi

A total of 20 terminal shoots per tree from four trees per treatment group were tagged and monitored. The emergence time of the “whitish millet” stage; appearance of white, millet-sized floral primordia at the shoot apex; percentage of flowering shoots, panicle axis, and lateral branches clearly visible; percentage of pure flowering shoots; and inflorescence length and width were recorded during full bloom for terminal shoots at different maturity stages. The flowering rate was calculated as the percentage of tagged terminal shoots. To minimize observer bias, all phenological assessments were performed independently by two trained observers, and the average values were used for subsequent analysis.

### 4.3. Quantification of Leaf Carbohydrates

Soluble sugar profiles in litchi leaves were determined following a protocol adapted from Yang [39]. Briefly, cryogenic liquid nitrogen was used to homogenize frozen leaf samples. A 0.3 g portion of the pulverized tissue was suspended in 6 mL of 90% (*v*/*v*) ethanol, followed by a 20 min thermal incubation in an 80 °C water bath. The resulting slurry was centrifuged at 4000 rpm for 10 min under ambient conditions. The remaining residue underwent an identical re-extraction step. Supernatants from both extractions were pooled and concentrated to dryness using a rotary evaporator (RapidVap, Labconco, Kansas City, MO, USA).

The dried extract was reconstituted in double-distilled water, then clarified via centrifugation at 13,000× *g* for 10 min. To eliminate impurities, the supernatant was passed through a Sep-Pak^®^ C18 cartridge (1 cc/100 mg; Waters Corp., Milford, MA, USA). Sugar separation and quantification were carried out on an Agilent 1200 HPLC system (Agilent Technologies, Waldbronn, Germany) integrated with a Transgenomic CARB Sep Coregel 87C column accompanied by a matching guard cartridge. The analytical column and the refractive index detector were held at 80 °C and 40 °C, respectively. Chromatographic runs were executed with an injection volume of 10 μL using deionized water as the mobile phase at a constant flow rate of 0.4 mL min^−1^. The sugar standards (sucrose, glucose, quebrachitol, and fructose) were purchased from Sigma-Aldrich, St. Louis, MO, USA. Target sugars were identified by aligning retention times with authentic standards and quantified against corresponding calibration curves. Standard curves were prepared using a series of concentrations ranging from 1 to 40 mg/g, and good linearity was obtained for all sugars, with coefficients of determination R^2^ > 0.999 for each standard curve. For each time point, all litchi tissue samples were analyzed in at least three independent biological replicates.

### 4.4. Real-Time PCR Analysis

Genes associated with energy metabolism were retrieved from the litchi genome database, Available online: http://121.37.229.61:82/ (accessed on 2 August 2022). Specific primer pairs were synthesized using Primer 5.0 software (Supplementary Table S1). Total RNA was extracted from the leaves of mature terminal shoots and expanding terminal shoots at each sampling time point (0, 1, 7, 20, 40, 60, and 68 DAT) as described in Section 2.1. Quantitative real-time PCR was performed on an ABI 7500 Real-Time PCR System (Applied Biosystems, Foster City, CA, USA). The thermocycling conditions comprised an initial denaturation at 95 °C for 5 min, followed by 40 cycles of denaturation at 95 °C for 10 s, annealing at 55 °C for 30 s, and elongation at 72 °C for 30 s, with a final melting curve analysis to confirm reaction specificity. All reactions were executed in at least three independent biological replicates, with data presented as the mean ± standard error (SE). Target gene expression levels were normalized to LcActin (GenBank accession no. HQ615689), a well-validated reference gene exhibiting stable expression across litchi cultivars and under abiotic stresses [40]. Relative transcript abundances were quantified using the 2^–∆∆CT^ method [41]. Statistical significance between groups was determined using one-way ANOVA followed by a *t*-test for multiple comparisons, with significance levels set at *p* < 0.05 and *p* < 0.01. For each treatment group and time point, total RNA was extracted from three independent biological replicates, each consisting of pooled leaf tissue from at least five terminal shoots collected from different trees. For each biological replicate, qRT-PCR was performed with three technical replicates per primer pair.

### 4.5. Dual-Luciferase Reporter Assay

The transcriptional activity of LcSPLs on the *LcFT1* promoter was evaluated using a dual-luciferase system according to Hellens [42]. The CDS of LcSPLs and the *LcFT1* promoter were cloned into pGreen II 0029 62-SK (effector) and pGreen II 0800-LUC (reporter) vectors, respectively. The genes and primer pairs used for Dual-Luciferase Reporter Assay are listed in Table S2. Agrobacterium tumefaciens (GV3101) cells carrying the constructs were prepared in induction buffer (10 mM MES, 10 mM MgCl_2_, 200 μM acetosyringone, pH 5.6) to OD_600_ = 0.8. Mixtures of effector and reporter strains were co-infiltrated into leaves of 3-week-old Nicotiana benthamiana, with empty effector vector co-infiltrations as negative controls. After 3 days of growth at 23 °C, relative luciferase activities (LUC/REN) were measured using a GloMax 20/20 Luminometer (Promega Corporation, Madison, WI, USA). Assays included three biological replicates and were repeated twice independently.

### 4.6. Ectopic Overexpression of Litchi Genes in Arabidopsis

To generate overexpression constructs, the full-length coding sequences (CDSs) of *LcSPL1/3/9/10* were inserted into the pCAMBIA1302 vector via homologous recombination, the genes and primer pairs are listed in Table S2. The validated recombinant plasmids were subsequently transformed into Agrobacterium tumefaciens strain GV3101. *Arabidopsis thaliana* ecotype Columbia-0 (Col-0) plants were transformed using the floral dip method. Transgenic T1 seeds were surface-sterilized and selected on Murashige and Skoog (MS) agar medium supplemented with hygromycin. Seven-day-old resistant seedlings were transplanted into a soil mixture (nutrient soil–vermiculite–perlite = 4:1:1, *v*/*v*/*v*) and maintained in a growth chamber at 23 °C under long-day conditions. For each construct, at least three independent T3 homozygous lines were selected for phenotypic analysis. Flowering time was scored as the number of rosette leaves at bolting and the number of days from sowing to the appearance of the first visible floral bud. At least 9 plants were scored per line.

### 4.7. Data Processing and Statistical Analysis

Experimental data were processed using Microsoft Excel, and figures were generated with Origin 2021 software (OriginLab Corp., Northampton, MA, USA). Biological flowering parameters were subjected to one-way analysis of variance (ANOVA). Differences among treatment means were determined using a *t*-test for multiple comparisons at a significance level of *p* < 0.05 via SPSS 21.0 (IBM Corp., Armonk, NY, USA). Linear relationships between variables were evaluated using Pearson’s correlation analysis.

## 5. Conclusions

In conclusion, this study provides pivotal physiological and molecular insights into how terminal shoot maturity dictates low-temperature floral induction in litchi. We demonstrate that immature terminal shoots represent a major physiological barrier to floral initiation due to deficient carbohydrate accumulation, impaired T6P signaling, and unfavorable transcriptional outputs. Specifically, terminal shoot maturation establishes an essential metabolic foundation characterized by the accumulation of soluble sugars and the sustained activation of *LcTPS1/3/4* genes. At the genetic level, mature shoots selectively enrich flower-promoting transcription factors, among which *LcSPL1*, *LcSPL3*, and *LcSPL10* act as critical positive regulators. Mechanistically, these LcSPLs transcriptionally activate the promoter of the primary florigen gene *LcFT1*, effectively tipping the *LcFT1/LcTFL1* balance toward reproductive transition at the shoot apical meristem. In contrast, immature shoots maintain elevated expression of the floral repressor *LcTFL1* and the transcriptional suppressor *LcSPL9*, suppressing *LcFT1* transactivation and perpetuating vegetative growth even under favorable chilling conditions.

Overall, our findings uncover an integrated “metabolism–LcSPL–*LcFT1*” regulatory network by which shoot maturity conditions environmental responsiveness. These theoretical advancements not only resolve a long-standing question in litchi floral biology but also offer practical guidance for horticultural practices aimed at accelerating terminal shoot maturation to mitigate alternate bearing and achieve reliable yield in perennial fruit crops.

**Figure 9:**
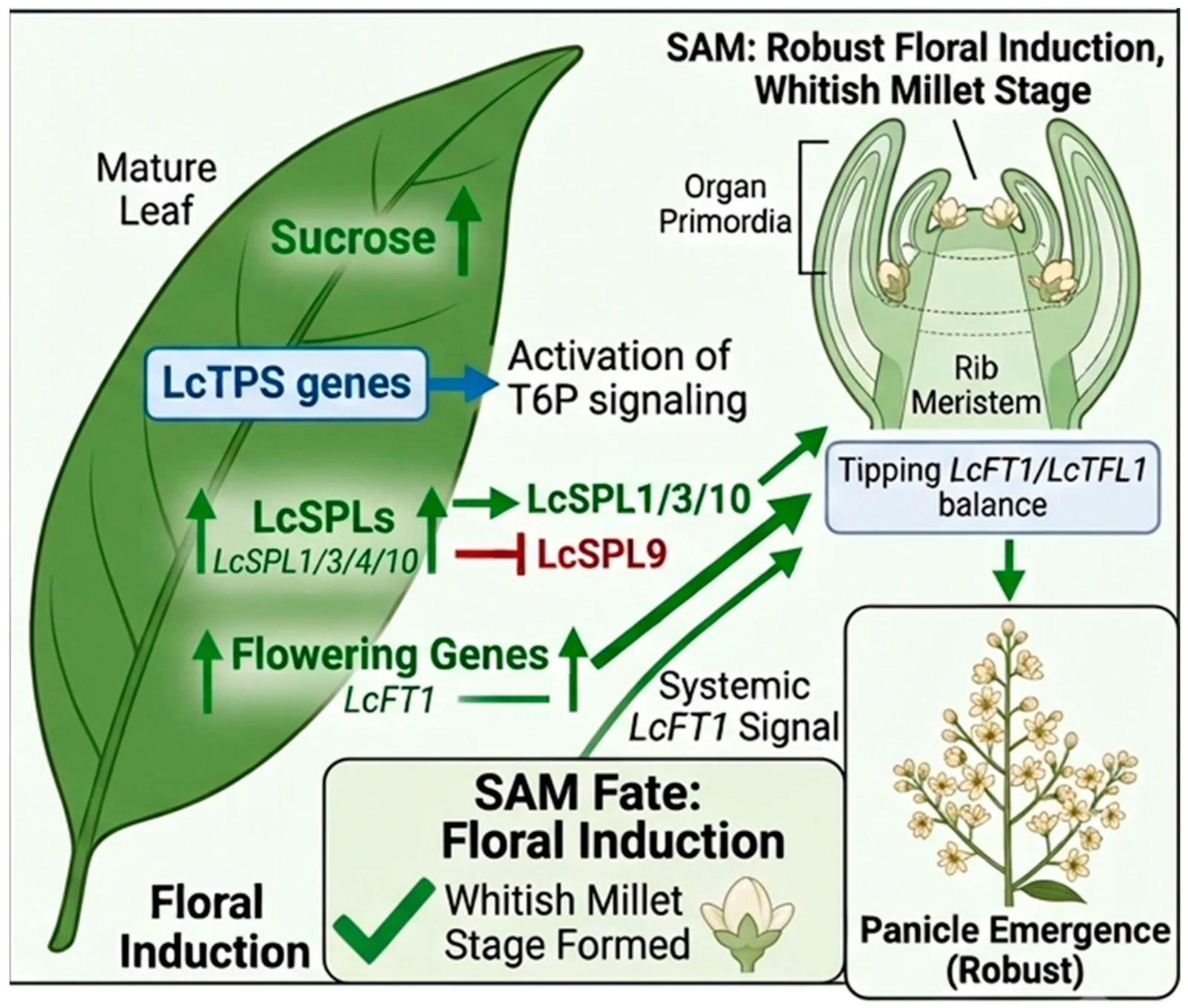
Proposed regulatory model of terminal shoot maturity on low-temperature floral induction in litchi. In mature leaves, chilling-induced sucrose accumulation and *LcTPS* expression activate T6P signaling, promoting *LcSPL1/3/10* while suppressing *LcSPL9*. LcSPL1/3/10 transactivates *LcFT1*, sending systemic signals to the SAM to tip the *LcFT1/LcTFL1* balance and driving “whitish millet” formation and robust panicle emergence.

Based on our physiological, biochemical, and molecular findings, we propose a hypothetical working model to illustrate how terminal shoot maturity conditions low-temperature responsiveness and dictates floral induction in litchi (Figure 9). In mature litchi leaves, winter chilling induces substantial sucrose accumulation and sustained transcription of *LcTPS1/3/4*, activating T6P signaling to establish a high-energy metabolic status. This physiological state selectively upregulates the flowering-promoting transcription factors LcSPL1, LcSPL3, and LcSPL10, which transcriptionally activate the promoter of the core florigen gene *LcFT1* while repressing the immature shoot-enriched suppressor LcSPL9. The resulting systemic *LcFT1* signal translocates to the SAM, tipping the *LcFT1/LcTFL1* transcriptional balance to drive SAM morphological reorganization, form the “whitish millet” stage, and ultimately enable robust panicle emergence under subsequent ambient temperatures.

## Figures and Tables

**Table 1:** Flowering statistics of litchi terminal shoots at different maturity stages after low-temperature induction.

Group	“Whitish Millet” Stage (d)	Flowering Rate (%)	Pure Flowering Rate (%)	Inflorescence Length (cm)	Inflorescence Width (cm)
Expanding	73.46 ± 10.78	14.51 ± 5.31	0	7.50 ± 1.77	4.20 ± 1.04
Mature	70.33 ± 11.62	84.62 ± 15.38 **	62.22 ± 23.43 **	14.90 ± 1.06 *	9.59 ± 0.76 *

Note: * indicates a significant difference in the same column at the *p* < 0.05 level; ** indicates a significant difference in the same column at the *p* < 0.01 level.

**Table 2:** Flowering time of T2 plants transformed with *35S::LcSPL1/3/9/10*.

Plant Genotype	Number of Plant	Days to Flowering	Number of Rosette Leaves
Wt (col)	6	28.39 ± 0.52 a	14.85 ± 0.46 a
35S::*LcSPL1*	6	23.52 ± 1.36 b	10.46 ± 0.75 b
35S::*LcSPL3*	6	22.79 ± 1.58 b	11.16 ± 1.31 b
35S::*LcSPL9*	6	29.62 ± 1.89 a	14.65 ± 0.98 a
35S::*LcSPL10*	6	23.95 ± 2.18 b	11.67 ± 1.22 b

Different lowercase letters in the same column indicate significant differences at *p* < 0.05.

## Data Availability

The original contributions presented in this study are included in the Supplementary Materials.

## Supplementary Materials

The following supporting information can be downloaded at: https://www.mdpi.com/article/10.3390/plants15182829/s1. Table S1: Genes and primer pairs used for quantitative real-time PCR; Table S2. Genes and primer pairs used for Dual-Luciferase Reporter Assay and Ectopic Overexpression.
