# Supplementary material for: Terminal Shoot Maturity Orchestrates LcSPL–LcFT1 Signaling to Dictate Low-Temperature Floral Induction in Litchi (Litchi chinensis Sonn.)
Source: Plants (Basel). 2026 Sep 16;15(18):2829. doi: 10.3390/plants15182829 (PMC13610586; doi:10.3390/plants15182829)
Supplement: Supplementary file 1 [file plants-15-02829-s001.zip › plants-4530191-supplementary.pdf]

Table S1. Genes and primer pairs used for quantitative real-time PCR

Gene name	Gene ID	Forward primer (5’→3’)	Reverse primer (5’→3’)
LcFT1	LITCHI002331	GCCGCGGTCTACTTTAATAGC	TCTTTGCTAGCTTTGACATGC
LcFT2	LITCHI002334	CTTCAACTGCCAGAGGGAGA	TCTTTGCTAGCTTTGACATGC
LcFD	LITCHI010080	CTACCTCTGTGAAACGCTTGG	GACTGCCCCTCTCAGCTTTTA
LcTFL1	LITCHI018838	GGACCAATTCAGCACCAAAG	GTAGTGAGGAGGGGTTGCTCT
LcActin	HQ615689.1	ACCGTATGAGCAAGGAAATCACTG	TCGTCGTACTCACCCTTTGAAATC
LcTPS1	LITCHI012687	CCTCGTACAAATGCCCGATT	GCGCCTAACAATGATTCG
LcTPS2	LITCHI028930	TACCAGGGGAGAGAAGACCAT	GCCGTCCACTATTACCGTTCT
LcTPS3	LITCHI023128	AGTGGATCCAAGGTAGGTCGT	ATTGTAGGCCACCAAACACC
LcTPS4	LITCHI021186	GGGAGTGATTAGACTCGTCCAG	TCTGCAAGACATTGGACTGC
LcTPS5	LITCHI020455	TAGGTTGATTCAGGGCTTGG	GAAGCACCCAGCTCAGCTTAT
LcTPS6	LITCHI005877	GCAGCTGCACATCGTAAATC	CCATATGTGCCCCTTCTAACAC
LcTPS7	LITCHI031282	CACTCTGGATGTTATGGCAGTG	CTCCGACGAAACAAAAGCAG
LcTPS8	LITCHI028829	CTTGCAGAAGCCTCAGACC	CATTCATAGGCCAGCATCAG
LcTPS9	LITCHI004984	AGGCCTTGCAACAGCTTC	CGGAAAGGCTCCAACAAGTA
LcSPL1	LITCHI013083	AATGCCATTCCAAGGGAATAC	ATGAAAGGAACAGAACATGC
LcSPL3	LITCHI013272	CCATGGAGAAGGCTCAAACTG	GCCGGCTTGGTGCATAAT
LcSPL4	LITCHI004647	AACTGCGCTTGCTTTCAGTT	CGCAATCAGCTAGCAATCCT
LcSPL6	LITCHI025416	ACATTGCATCGCCGACAGGAC	GTACGATTCTCCACGATGAGC
LcSPL9	LITCHI004652	CCTGGACTTCCTTCAGGAGAA	AAGACCTGATGCTCGGTTTC
LcSPL10	LITCHI021016	CGGCAACTAAAGGCTGCAAT	AGCCATGAGCATTCCTGTTG
LcSPL12	LITCHI019149	GGTGCGACACAAGATTGTCA	CACAGTCTGAGGCATGTTGGT
LcSPL13	LITCHI014255	ACCATGCAAGGGTTATCAGG	GGAATTGTGGCCTGTTAGGAC
LcSPL14	LITCHI014612	CTTCTGAGGACCATTGCTCTG	GGCTTAAGCTGATCCCAGATG
LcSPL17	LITCHI023329	ATGGTGGCAGTAGCCAGAAAG	ATCTCCTGAGTCTCGGCTTGT
LcActin	HQ615689.1	ACCGTATGAGCAAGGAAATCACTG	TCGTCGTACTCACCCTTTGAAATC

Table S2. Genes and primer pairs used for Dual-Luciferase Reporter Assay and Ectopic Overexpression

Gene name	Target	Forward primer (5’→3’)	Reverse primer (5’→3’)
LcFT1pro-LUC	DLR assay	AggtaccCCTAGCCTATTCTTTGATAT	AggatccCTATCACTCGGCCAACCACA
LcSPL1-SK	DLR assay	AggatccATGGATTGCACAAGCCTTGAA	AgaattcTTATCTGATCTGGGAACGCTTG
LcSPL3-SK	DLR assay	AggatccATGGACACAAGCAAAGCTGAAG	AgaattcTCAGTTTGAGCCTTCTCCATGG
LcSPL4-SK	DLR assay	AggatccATGGATGGCAAAGGCAAGAA	AgaattcTTATCTGATCTGGAAGTGTTTGA
LcSPL6-SK	DLR assay	AggatccATGGAGAATGGTAATAACAGTAA	AgaattcCATCCAGAGACTTGGGTACAC
LcSPL9-SK	DLR assay	AggatccATGGAACTGGGTTCAGGCTCT	AgaattcTTAAAGTGACCAGTTCATCTGCG
LcSPL10-SK	DLR assay	AggatccATGGAGTCTTGGAGCTATGACTCT	AgaattcTTATGAGGTCAGGAAACAGCAA
LcSPL12-SK	DLR assay	AggatccATGGAGTGGAATGGGAAAATCC	AgaattcTCAGTTCATATGATTAAAATAAAA
LcSPL13-SK	DLR assay	AggatccATGGAGTCTTCTTGGAGCTATGTG	AgaattcTCAGAGGTGGCAGAAAGCATC
LcSPL14-SK	DLR assay	AggatccATGGAATGCATGTCAGTGAATAAA	AgaattcCTACTCCCACTGAAAGGGAATGG
LcSPL17-SK	DLR assay	AggatccATGGAATCATTAGCATCTGGTTCTT	AgaattcTCACTCATCAGGGCCAATGTG

LcSPL1-OE	*OE* assay	AgaattcATGGATTGCACAAGCCTTGAAG	AggatccTTATCTGATCTGGGAACGCTTG
LcSPL3-OE	*OE* assay	AgaattcATGGACACAAGCAAAGCTGAAG	AggatccTTAAAGTGACCAGTTCATCTGCG
LcSPL9-OE	*OE* assay	AgaattcATGGAACTGGGTTCAGGCTC	AggatccTCAGTTTGAGCCTTCTCCATGG
LcSPL10-OE	*OE* assay	AgaattcATGGAGTCTTGGAGCTATGAC	AggatccTTATGAGGTCAGGAAACAGCAA
